# Complete Mitochondrial Genomes of *Nedyopus patrioticus*: New Insights into the Color Polymorphism of Millipedes

**DOI:** 10.3390/cimb46030159

**Published:** 2024-03-15

**Authors:** Gaoji Zhang, Tangjun Xu, Yukun Chen, Wei Xu, Yinuo Wang, Yuanyuan Li, Fuyuan Zhu, Hongyi Liu, Honghua Ruan

**Affiliations:** 1 College of Life Sciences, Nanjing Forestry University, Nanjing 210037, China; zhanggaoji@njfu.edu.cn (G.Z.); xutangjun@njfu.edu.cn (T.X.); 2932797114@njfu.edu.cn (Y.C.); xuwei2001@njfu.edu.cn (W.X.); 17766100299@163.com (Y.W.); fyzhu@njfu.edu.cn (F.Z.); 2 College of Ecology and the Environment, Nanjing Forestry University, Nanjing 210037, China; lee-oo@foxmail.com (Y.L.); hhruan@njfu.edu.cn (H.R.)

**Keywords:** color polymorphism, mitochondrial genomes, *Nedyopus patrioticus*, phylogenetic analysis

## Abstract

There has been debate about whether individuals with different color phenotypes should have different taxonomic status. In order to determine whether the different color phenotypes of *Nedyopus patrioticus* require separate taxonomic status or are simply synonyms, here, the complete mitochondrial genomes (mitogenomes) of two different colored *N. patrioticus*, i.e., red *N. patrioticus* and white *N. patrioticus*, are presented. The two mitogenomes were 15,781 bp and 15,798 bp in length, respectively. Each mitogenome contained 13 PCGs, 19 tRNAs, 2 rRNAs, and 1 CR, with a lack of *trnI*, *trnL2*, and *trnV* compared to other Polydesmida species. All genes were located on a single strand in two mitogenomes. Mitochondrial DNA analyses revealed that red *N. patrioticus* and white *N. patrioticus* did not show clear evolutionary differences. Furthermore, no significant divergence was discovered by means of base composition analysis. As a result, we suggest that white *N. patrioticus* might be regarded as a synonym for red *N. patrioticus*. The current findings confirmed the existence of color polymorphism in *N. patrioticus*, which provides exciting possibilities for future research. It is necessary to apply a combination of molecular and morphological methods in the taxonomy of millipedes.

## 1. Introduction

The existence of two or more distinctly colored phenotypes among individuals of an interbreeding population is known as color polymorphism [1]. Color polymorphism is common in many animals, occurring from invertebrates to vertebrates [2,3,4]. It is an ideal model system to investigate and understand fundamental evolutionary processes [5]. However, whether different phenotypes truly require separate taxonomic status, or whether these co-occur and belong to a single taxon, has been a subject of debate and requires more biological information to provide a basis for establishing the answer [1].

The mitochondrial genome (mitogenome) is widely used in the research of the evolutionary origin and genetic diversity of organisms due to its fast evolution rate, simplified structure, and efficient genetic information [6,7,8]. The mitogenome of animals is usually a circular, double-stranded molecule, typically containing a standard set of 13 protein-coding genes (PCGs), two ribosomal RNA genes (rRNAs), 22 transfer RNA genes (tRNAs), and one control region (CR) [9]. This set of 37 genes is conserved across bilaterian metazoans, with only a few exceptions, such as a small number of genes lost in some derived groups [10]. In arthropods, the mitogenome exhibits diverse structures, and aberrant genomic systems are present. For example, each tRNA gene has been severely truncated in some species of the order Diptera [11]. Additionally, the mitogenomes of some species in the order Anoplura have been observed to split into several chromosomes [12]. Previous studies have found differences in the mitogenome among species, subspecies, and geographic populations of invertebrates, which can be used to explore the genetic diversity and evolution of invertebrates [13,14].

The tribe Nedyopodini is one of the most characteristic elements in the paradoxosomatid fauna of east and southeast Asia [15]. However, the tribe Nedyopodini is perhaps one of the most confused tribes of Paradoxosomatidae in taxonomy. The existing descriptions of its genera, species, and subspecies are often very poor. Even a few actual morphological keys are too shallow to be meaningful [16]. In order to better understand the classification relationship of the tribe Nedyopodini, a classification method based on its mitogenome should be adopted to enhance the results of morphological methods. Research on the complete mitogenome can increase the opportunity to identify taxonomic relationships [17].

*Nedyopus patrioticus* belongs to the class Diplopoda, order Polydesmida, family Paradoxosomatidae [18]. So far, there are two subspecies recognized in *N. patrioticus*: *N. patrioticus patriotocus* and *N. patrioticus unicolor* [15]; however, there are very few descriptions available of them. The homology of the two subspecies is also disputed. These two color variations of *N. patrioticus* can be used as an example to study the taxonomic relationships of color-polymorphic species.

In this study, we present the complete mitogenomes of red *N. patrioticus* and white *N. patrioticus*. We try to verify the taxonomic relationship of these two color variations of *N. patrioticus* based on their mitogenomes. The results of this study provide new insights into the color polymorphism of *N. patrioticus* and the phylogenetic relationships of Diplopoda. Our study makes a certain contribution to the determination of the taxonomic relationships of color-polymorphic species. Our results could also lay the foundation for research on color polymorphism.

## 2. Materials and Methods

### 2.1. Sample Collection and DNA Extraction

A total of 17 individuals, including 10 red *N. patrioticus* and 7 white *N. patrioticus*, used in this paper were captured on 24 May 2023 in the moist section of a deciduous forest on Mufu Mountain (32°7′ N, 118°47′ E) in Nanjing, Jiangsu, China. After species diagnosis performed based on morphological features given in previous research [16] and the distribution area provided by the Global Biodiversity Information Facility website (GBIF, available at https://www.gbif.org, accessed on 9 October 2023) [19], the specimens were stored in a −80 °C refrigerator at the Nanjing Forestry University Animal Molecular Evolution Laboratory. Due to the lack of relevant previous research, we could not determine which subspecies the red *N. patrioticus* and white *N. patrioticus* belong to. The collection of the specimens was reviewed and approved by Nanjing Forestry University. The specimens used in this study were collected and studied in accordance with Chinese laws. Total genomic DNA of two samples was extracted using a FastPure Cell/Tissue DNA Isolation Mini Kit (Vazyme, Nanjing, China), and stored at −20 °C for the follow-up investigation.

### 2.2. Sequence Analysis

Library construction and sequencing were carried out using the Illumina platform (Personal, Shanghai, China) with an insert size of 300 bp (about 4 Gb of raw data). To generate clean data, low-quality sequences were removed. The mitogenome of *Asiomorpha coarctata* (accession no. KU721885.1) was used as a template for assembly using Geneious Prime 2023 software [20]. The medium sensitivity/speed option was used for the assembly. Two consensus sequences were generated with a 50% base call threshold, obtaining the complete mitogenomes of red *N. patrioticus* and white *N. patrioticus*.

The preliminary examination of the two mitogenomes was conducted using DNASTAR Lasergene 7.1 and the MITOS Web Server (available at https://usegalaxy.eu/root?tool_id=tooshed.g2.bx.psu.edu%2Frepos%2Fiuc%2Fmtos%2Fmtos%2F1.1.1%20galaxy0, assessed on 20 October 2023) for sequence alignment and gene recognition [21,22,23]. The MITOS Web Server was utilized to locate RNA genes. The PCGs were predicted using both MITOS and the CD-search tool on the NCBI website (available at https://www.ncbi.nlm.nih.gov/, assessed on 22 October 2023). The correct mitogenomes were submitted to GenBank (accession numbers: OR755973.1 for red *Nedyopus patrioticus*; OR777861.1 for white *Nedyopus patrioticus*). The composition skew was calculated based on the following formula: AT-skew = (A − T)/(A + T) and GC-skew = (G − C)/(G + C) [24]. MEGA X software was utilized to calculate the relative synonymous codon usage (RSCU) and the non-synonymous (Ka) and synonymous substitutions (Ks) [25]. The ggplot2 and aplot packages, implemented in R v.4.3.1, were employed to produce visual representations of the data [26]. The prediction of protein secondary structures was conducted using the GOR4 secondary structure prediction method (available at https://npsa-prabi.ibcp.fr/, accessed on 2 November 2023) [27].

### 2.3. Phylogenetic Analysis

A total of 24 species of Diplopoda with complete mitogenomes, representing 11 families and 7 orders, were included in the phylogenetic analyses (Table 1). Additionally, a Chilopoda species, *Cermatobius longicornis*, was utilized as an outgroup. Phylogenetic analyses were conducted using sequences of amino acids. All procedures were carried out using the PhyloSuite v1.2.3 software package [28]. Multiple sequence alignments were performed using MAFFT v7.313 “Normal” mode, and the sequence pruning of amino acids was executed using the “Protein” pattern of Gblock. With BIC as the standard criterion, partition analysis was performed for IQ-TREE and Mrbayes using ModelFinder’s edge-unlinked mode [29]. A Bayesian inference (BI) tree was reconstructed using MrBayes with four Markov Chain Monte Carlo chains (three hot chains and one cold chain) [30]. Two independent runs of 1,000,000 generations were conducted with sampling every 1000 generations, and the first 25% of samples were discarded as burn-ins to reduce simulation error. The best-fit models obtained using ModelFinder for MrBayes were as follows: partition 1 (*CYTB*, *ATP6*, *ATP8*, *COXII*, *COXIII*, *ND1*, *ND2*, *ND3*, *ND4*, *ND4L*, *ND5*, *ND6*), model mtREV+F+I+G4; partition 2 (*COXI*), model mtMAM+I+G4. A maximum likelihood (ML) tree was reconstructed using IQ-TREE with 1000 bootstrap replicates [31]. The best-fit models obtained using ModelFinder for IQ-TREE were as follows: partition 1 (*CYTB*, *ATP6*, *ATP8*, *COXII*, *COXIII*, *ND1*, *ND2*, *ND3*, *ND4*, *ND4L*, *ND5*, *ND6*), model mtZOA+F+R5; partition 2 (*COXI*), model LG+F+I+G4. Phylogenetic trees were visualized and edited using the Interactive Tree of Life Web Server (iTOL, available at https://itol.embl.de, accessed on 9 November 2023) [32].

## 3. Results and Discussion

### 3.1. Genome Organization and Composition

**Figure 1:**
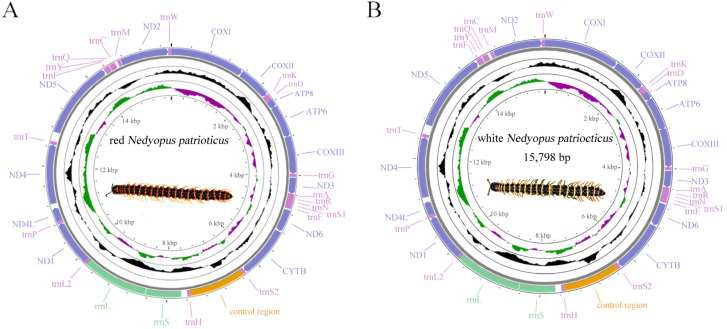
Circular map of the mitogenomes of red *Nedyopus patrioticus* (**A**) and white *Nedyopus patrioticus* (**B**). Yellow blocks: control region; green blocks: rRNAs; light purple blocks: tRNAs; dark purple blocks: PCGs.

The mitogenome lengths of red *N. patrioticus* and white *N. patrioticus* were 15,781 bp and 15,798 bp, respectively (Table 2 and Figure 1). These lengths were well within the range found in other species of Polydesmida. Each mitogenome contained 13 PCGs, 19 tRNAs, 2 rRNAs, and one CR, which was different from typical sets of genes found in invertebrate mitogenomes in terms of the lack of *trnI*, *trnL2*, and *trnV* [33]. The genes of both mitogenomes were all situated on the minor strand (N-stand), a characteristic shared with other species of Polydesmida [20,33].

The lengths of every gene in the two mitogenomes were mostly identical. The base composition of red *N. patrioticus* was A 25.11%, T 43.12%, G 23.21%, and C 8.54%, whereas the base composition of white *N. patrioticus* was A 25.21%, T 43.07%, G 23.20%, and C 8.52%. The base compositions of the two genomes were almost the same. Base composition analysis showed that the whole mitogenomes of Polydesmida species were biased towards A and T (Table 3), from 64.04% for *Appalachioria falcifera* to 75.11% for *Epanerchodus koreanus*, which was the same as in previous studies [33]. Additionally, the A + T% of PCGs, rRNAs, and tRNAs in Polydesmida species were also higher than the G + C%. Skewness analysis based on base composition was used to estimate the relative numbers of A to T and G to C. The results of skewness showed that the AT-skews of Polydesmida species were negative, and the GC-skews were positive, which was consistent with other millipedes [34]. The A + T% and skewness of red *N. patrioticus* and white *N. patrioticus* were all nearly identical. 

These two mitogenomes had two identical overlapping regions: one between *ATP8* and *ATP6* and the other between *rrnL* and *trnL1*. In addition, the mitogenome of white *N. patrioticus* had an extra overlapping region between *ATP6* and *COX3*. The longest overlapping region of the two mitogenomes was found between *rrnL* and *trnL1*, measuring 11 bp in length.

### 3.2. Protein-Coding Genes

The lengths of the PCGs in the two mitogenomes were both 10,767 bp, which was slightly lower than in other Polydesmida species [20,33]. The A + T content of Polydesmida species ranged from 63.08% (*A. falcifera*) to 73.87% (*E. koreanus*). In the mitogenome of red *N. patrioticus*, seven PCGs (*COXI*, *COXII*, *ATP8*, *COXIII*, *ND4L*, *ND4*, *ND5*) used ATG as the initiation codon; four PCGs (*ATP6*, *ND6*, *CYTB*, *ND1*) used ATA as the initiation codon; and two PCGs (*ND3*, *ND2*) used ATT as the initiation codon. In the mitogenome of white *N. patrioticus*, six PCGs (*COXI*, *ATP8*, *COXIII*, *ND4L*, *ND4*) used ATG as the initiation codon; four PCGs (*ATP6*, *ND6*, *CYTB*, *ND5*) used ATA as the initiation codon; and two PCGs (*ND3*, *ND2*) used ATT as the initiation codon. A nonstandard initiation codon GTA was observed in *ND1* in the mitogenome of white *N. patrioticus*. Unusual initiation codons have previously been reported in many animals, including *ND2* of *Sellanucheza jaegeri*, which starts with TTG, and *COXI* of *Botyodes diniasalis*, which starts with CGA [35,36]. The termination codons of the two mitogenomes are identical; 10 PCGs (*COXI*, *COXII*, *ATP8*, *ATP6*, *COXIII*, *ND6*, *CYTB*, *ND4L*, *ND4*, *ND2*) used TAN (TAA, TAG) as termination codon, and the other PCGs (*ND3*, *ND1*, *ND5*) used T as the termination codon. These special termination codons are also found in other arthropods [37], and these codons might be transformed into TAA or TAG for formal functions [38].

**Figure 2:**
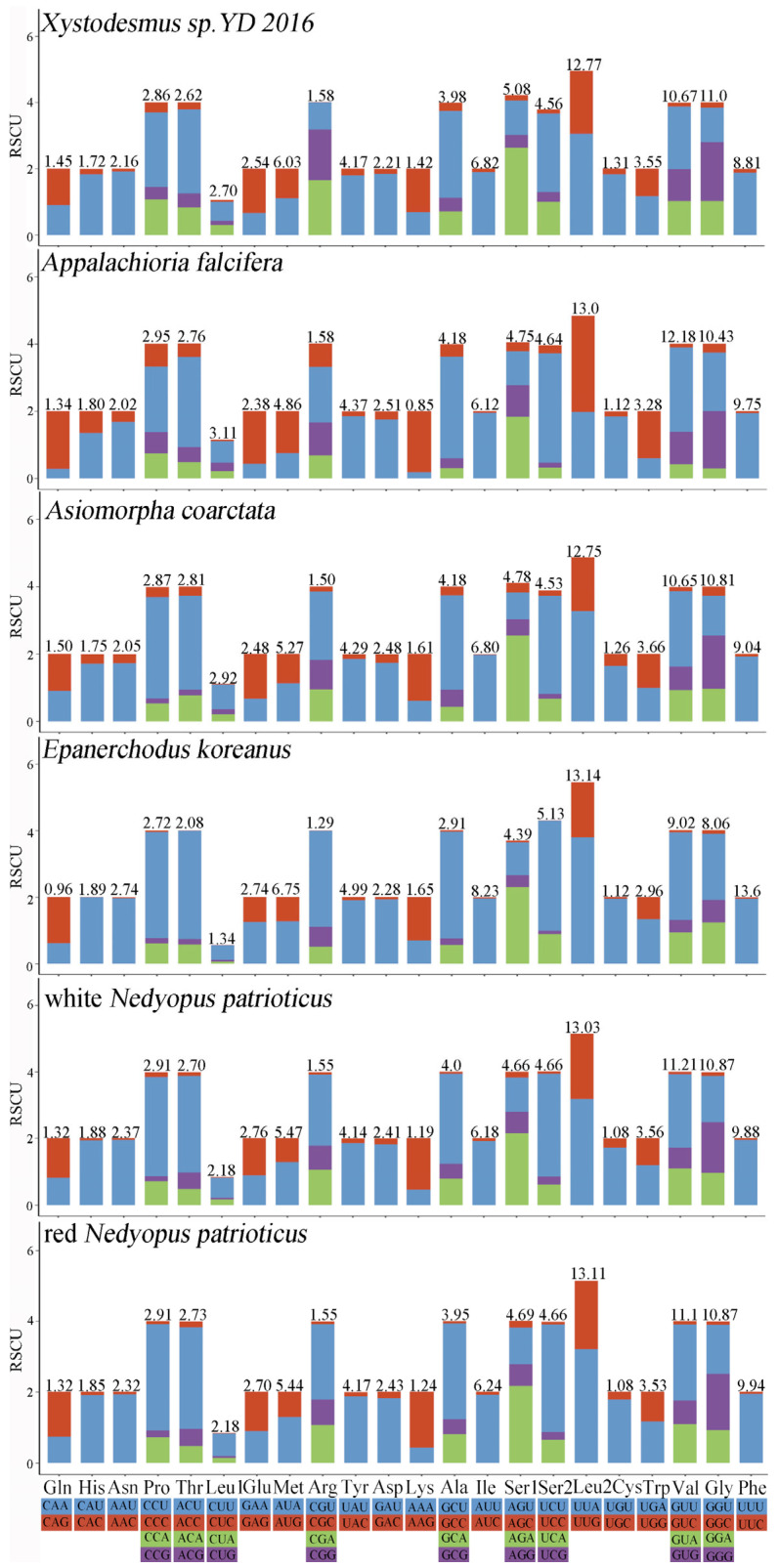
RSCU of Polydesmida. Different colors correspond to different third codons.

The RSCU values of six millipede species from Polydesmida were summarized to determine the frequency of synonymous codon usage (Figure 2). The three most commonly used amino acids were Leu2, Val, and Gly, whereas the three least used codon families were Cys, Arg, and His. Analogously, the biased use of A + T nucleotides was reflected in the codon frequencies. The usage of codons ending in A/U was significantly higher than that of codons ending in C/G, reflecting the strong AT bias of the third codon, a finding consistent with previous studies on the class Myriapoda [35,37].

**Figure 3:**
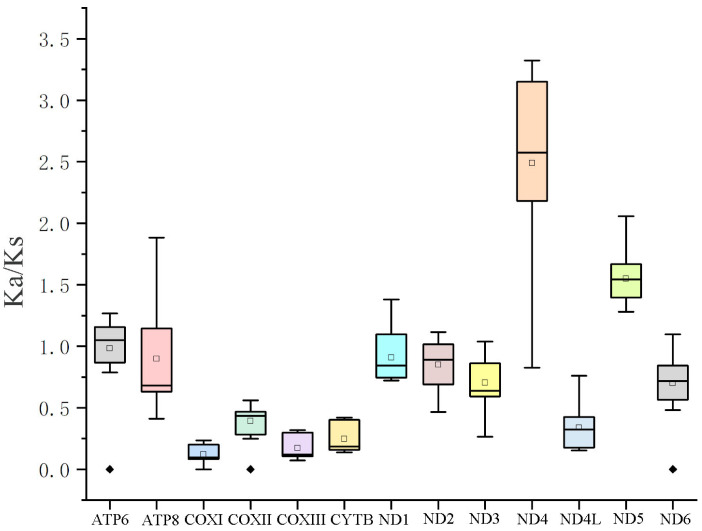
Ka/Ks values for the 13 PCGs of the order Polydesmida.

To analyze the evolutionary pattern of PCGs in polynemid species, the Ka/Ks values were assessed (Figure 3). Under the assumption of neutral protein-level evolution, the ratio of Ka to Ks should be equal, resulting in a Ka/Ks ratio of 1. A Ka/Ks ratio below 1 indicates the presence of purifying or stabilizing selection, which suggests a resistance to change. On the other hand, a ratio above 1 implies positive or Darwinian selection, which drives evolutionary change. The *ND4* gene (1.55) and the *ND5* gene (2.48) had an average Ka/Ks of more than 1, which suggests that the two genes experienced positive selection [39,40]. The *COXI* gene (0.17) had the lowest average Ka/Ks, suggesting a low evolution rate because of high selection pressure [39,40].

**Figure 4:**
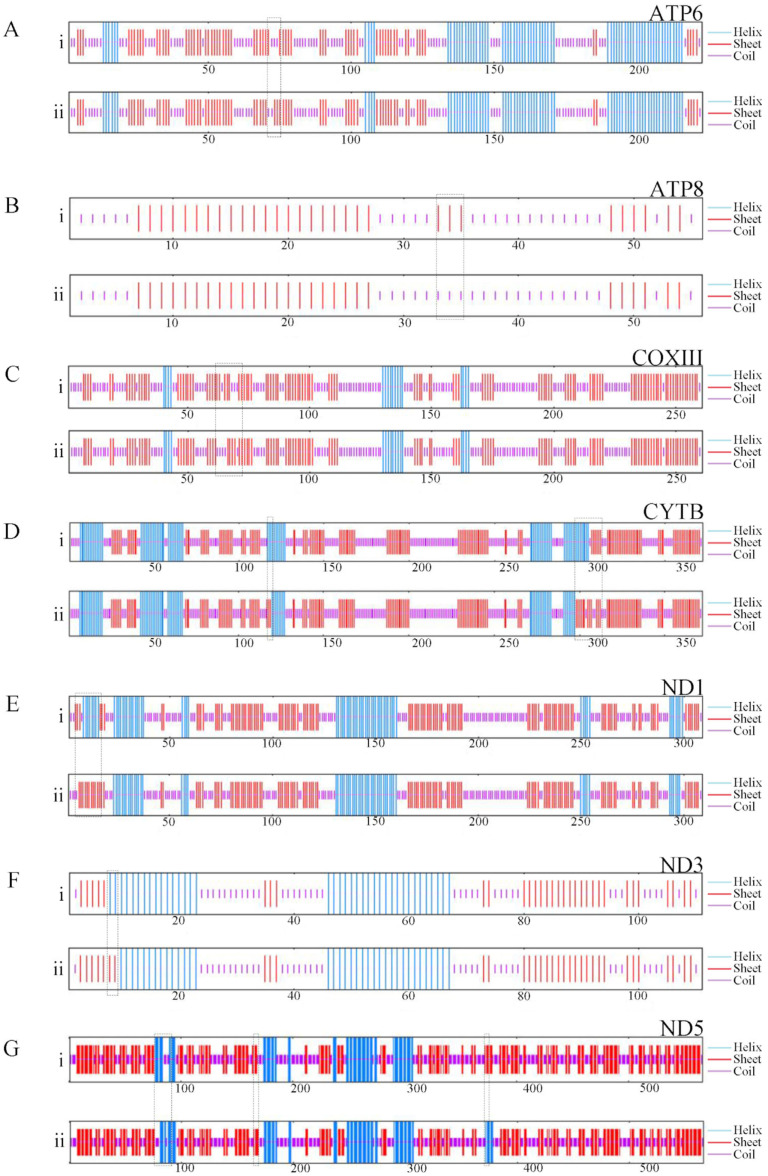
Effect of amino acid substitutions on the protein secondary structure encoded by mitogenomes of red *Nedyopus patrioticus* (**i**) and white *Nedyopus patrioticus* (**ii**). (**A**–**G**) Individual proteins; the area in the box represents the site of secondary structure change.

To enhance the examination of genetic variances between red *N. patrioticus* and white *N. patrioticus*, we employed GOR4 for the anticipation of the secondary structure of the polypeptide sequences (Figure 4). The location of variations in the secondary structure is marked by black block. Our findings revealed alterations in the secondary structure of seven proteins, with variations spanning from 1 position to 11 positions. The detailed analysis revealed that the variations in ATP6 (1 position), ATP8 (3 positions), and COXIII (1 position) lead to an increase in the random coil (Figure 4A–C). The variations in ND3 (2 positions) lead to an increase in the alpha helix (Figure 4F). The variations in CYTB (11 positions), ND1 (9 positions), and ND5 (8 positions) are more complex, including transformations in three secondary structures (Figure 4D,E,G). In summary, the genetic variance between red *N. patrioticus* and white *N. patrioticus* was small. This small genetic variation could be one reason for the color polymorphism.

### 3.3. rRNAs, tRNAs, and CR

*rrnS* and *rrnL* were located between *trnH* and *trnL1* (Table 2). The *rrnS* of two mitogenomes were both 737 bp in length. The length of *rrnL* was 1360 bp in red *N. patrioticus* and 1359 bp in white *N. patrioticus*.

There were 19 tRNAs in the two mitogenomes, respectively (Table 2), with a lack of *trnI*, *trnL2*, and *trnV* compared to other Polydesmida species [20,33]. The lengths of the two mitogenomes were 1203 bp and 1208 bp, which were comparatively smaller than those of other Polydesmida species, attributed to the lack of three tRNAs.

One CR was found between *trnS2* and *trnH* in the two mitogenomes, respectively (Table 2). The lengths of CR were 1168 bp and 1154 bp, with a difference of 14 bp, which was the primary reason for the variation in the lengths of the whole mitogenomes between red *N. patrioticus* and white *N. patrioticus*.

### 3.4. Gene Order

**Figure 5:**
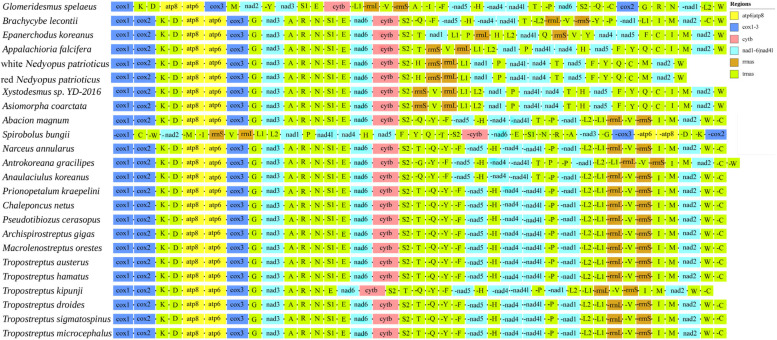
Gene arrangement of Diplopoda mitogenomes.

The arrangement of the mitogenome is considered a crucial tool for studying deep phylogenetic relationships because of its low rate of homoplasy [41]. Gene order arrangements were compared with mitogenome organization in other Diplopoda species (Figure 5). The gene order of the mitogenomes varies significantly in Diplopoda. For some Diplopoda species, mitochondrial gene order (MGO) patterns are shared at the family level (e.g., Spirostreptidae), whereas for other species, MGO patterns can differ within the same family. All genes were located on a single strand in two mitogenomes, which is consistent with the other species of Polydesmida [20,33]. Compared with other species of Polydesmida, three tRNAs *(trnV*, *trnL2*, and *trnI*) were lost in the two mitogenomes. And *trnH* underwent short-distance movements, resulting in the formation of *trnS2*-*trnH* gene clusters. The duplication–random loss (TDRL) model could potentially provide an explanation for this arrangement [42]. Based on this model, the replication process involves the duplication of specific DNA segments at homologous sites during replication, followed by their subsequent removal. This process ultimately leads to either the restoration of the original genomic organization or a rearrangement of the genome [43]. The gene order of Polydesmida is more susceptible to gene rearrangement between *trnS2* and *trnM* (Figure 5). To enhance our understanding of the evolutionary implications associated with gene arrangements in Diplopoda, it is essential to conduct further research on mitogenomes, covering a wider taxonomic range.

### 3.5. Phylogenetic Analysis

**Figure 6:**
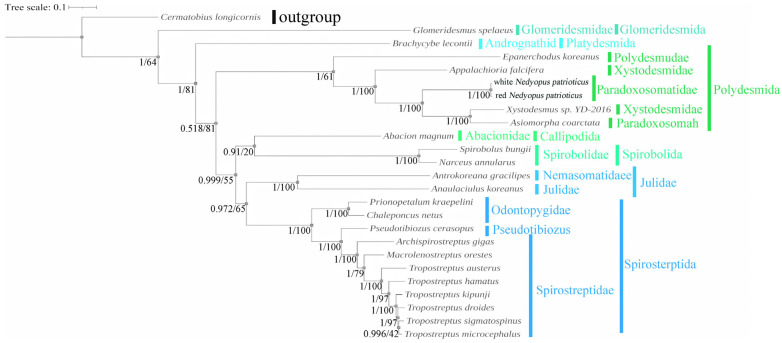
Amino acid-based phylogenetic tree of 24 Diplopoda for 13 PCGs. Numbers at nodes represent the posterior probability and bootstrap values for BI and ML analyses.

Because of the limited mitogenome sequences of Diplopoda species, we included only 24 species with credible annotations from 11 families of Diplopoda in the Phylogenetic analysis and selected one species in Chilopoda (*C. longicornis*) as an outgroup to root the phylogenetic trees. The results from both the BI and ML trees showed remarkable similarities and mutually supported each other (Figure 6). There is controversy about the sister-group relationship between *A. coarctata* and *Xystodesmus* sp. 2016. Yan Dong’s study suggested that *X*. sp. 2016 had a sister-group relationship with *A. falcifera* [20], while other studies proposed that *X*. sp. 2016 had a sister-group relationship with *A. coarctata* [35,44]. Our results provide strong support for the sister-group relationship between *X*. sp. 2016 and *A. coarctata* (posterior probability, PP = 1; bootstrap, BS = 100). This result reflects a potential flaw in the morphology-based species classification of Xystodesmidae and Paradoxosomah. The combination of molecular and morphological methods can lead to more accurate classification results. The phylogenetic analyses provided strong statistical support for the relationship between red *N. patrioticus* and white *N. patrioticus* (posterior probability, PP = 1; bootstrap, BS = 100). This result supports Attem’s hypothesis of conspecificity between *N. patrioticus patriotocus* and *N. patrioticus unicolor* [15]. The phylogenetic analyses provided strong support for the various families and orders within the Diplopoda. Our findings demonstrate that mitogenome sequences serve as effective molecular markers for examining the systematic relationships among Diplopoda species. However, it is important to note that our dataset included only 24 species, indicating its limited scope. To address the existing taxonomic debates and elucidate the higher-level phylogeny within Diplopoda species, it would be beneficial to expand sequencing efforts to encompass a greater number of taxa.

## 4. Conclusions

In summary, we show that the mitogenomes of two color variations of *N. patrioticus* exhibited high similarity in base composition, protein secondary structure, and gene order. In addition, there was a closer genetic relationship between red *N. patrioticus* and white *N. patrioticus* compared to other millipedes. Based on these foundations, we consider white *N. patrioticus* to be the same species as red *N. patrioticus*. In other words, *N. patrioticus patriotocus* and *N. patrioticus unicolor* are synonyms. Phylogenetic analysis has shown that mitogenomes can be a reliable tool for analyzing the phylogenetic relationships of Diplopoda species. This study is the first to report the complete mitogenomes of *N. patrioticus*, which will further enhance our understanding of the genetics, evolution, and taxonomy of the tribe Nedyopodini. In addition, previous studies have shown that different phenotypes exhibited due to color polymorphism may also belong to a synonym [1,45]. Our results indicate that classifying species with color polymorphism solely based on morphological characteristics is imperfect. It is necessary to apply a combination of molecular and morphological methods in the taxonomy of millipedes. In addition, this study has also demonstrated the necessity of integrating molecular and morphological methods in the taxonomy of millipedes.

Since mitochondrial genes serve the mitochondria themselves and their own protein synthesis and do not directly influence the expression of genes related to pigment composition in millipedes, our study is unable to explore the intricacies of biochemical genetics to reveal the molecular mechanisms of the inheritance of traits such as color variation. In order to explore the causes of color polymorphism and delve deeper into the intricacies of biochemical genetics, further studies based on nuclear data are needed.

## Figures and Tables

**Table 1:** The mitogenomes used in phylogenetic analyses.

Class	Order	Family	Species	Length (bp)	Accession No.
Dilopoda	Callipodida	Callipodidae	*Abacion magnum*	15,160	JX437062.1
	Glomeridesmida	Glomeridesmidae	*Glomeridesmus spelaeus*	14,863	MH590615.1
	Julida	Nemasomatidae	*Antrokoreana gracilipes*	14,747	DQ344025.1
		Julidae	*Anaulaciulus koreanus*	14,916	KX096886.1
	Playtdesmida	Andrognathidae	*Brachycybe lecontii*	15,115	JX437064.1
	Polydesmida	Paradoxosomatidae	*Asiomorpha coarctata*	15,644	KU721885.1
			*red Nedyopus patrioticus*	15,814	OR755973.1
			*white Nedyopus patrioticus*	15,798	OR777861.1
		Polydesmidae	*Epanerchodus koreanus*	15,581	MT898420.1
		Xystodesmidae	*Appalachioria falcifera*	15,282	JX437063.1
			*Xystodesmus* sp. YD-2016	15,791	KU721886.1
	Spirobolida	Spirobolidae	*Narceus annularus*	14,868	AY055727.1
			*Spirobolus bungii*	14,879	MT767838.1
	Spirostreptida	Odontopygidae	*Chaleponcus netus*	15,093	MT394513.1
			*Prionopetalum kraepelini*	15,114	MT394524.1
		Spirostreptidae	*Archispirostreptus gigas*	15,177	MT394525.1
			*Macrolenostreptus orestes*	15,367	MT394512.1
			*Pseudotibiozus cerasopus*	15,121	MT394506.1
			*Tropostreptus austerus*	15,261	MT394523.1
			*Tropostreptus droides*	15,172	MT394522.1
			*Tropostreptus hamatus*	15,156	MT394508.1
			*Tropostreptus kipunji*	15,170	MT394503.1
			*Tropostreptus microcephalus*	15,169	MT394516.1
			*Tropostreptus sigmatospinus*	15,176	MT394504.1
Chilooda	Lithobiomorpha	Henicopidae	*Cermatobius longicornis*	16,833	KC155628.1

**Table 2:** General features of the mitogenomes of red *Nedyopus patrioticus* and white *Nedyopus patrioticus*.

Gene	Location		Length (bp)	Intergenic Region	Codon		Stand
	From	To			Start	Stop	
*COXI*	1/1	1533/1533	1533/1533		ATG/ATG	TAG/TAG	N/N
*COXII*	1541/1541	2218/2218	678/678	7/7	ATG/ATG	TAA/TAA	N/N
*trnK*	2219/2219	2281/2281	63/63				N/N
*trnD*	2282/2282	2351/2351	70/70				N/N
*ATP8*	2352/2352	2522/2522	171/171		ATG/ATG	TAG/TAG	N/N
*ATP6*	2519/2519	3190/3190	672/672	−5/−5	ATA/ATA	TAA/TAA	N/N
*COXIII*	3192/3183	3968/3968	786/786	1/−9	ATG/ATG	TAA/TAA	N/N
*trnG*	3969/3969	4032/4032	64/64				N/N
*ND3*	4048/4048	4381/4381	334/334	15/15	ATT/ATT	T/T	N/N
*trnA*	4382/4382	4444/4444	63/63				N/N
*trnR*	4445/4445	4507/4507	63/63				N/N
*trnN*	4508/4508	4575/4575	68/68				N/N
*trnS1*	4576/4576	4634/4634	59/59				N/N
*trnE*	4637/4637	4697/4697	61/61	2/2			N/N
*ND6*	4728/4728	5195/5195	468/468	30/30	ATA/ATA	TAA/TAA	N/N
*CYTB*	5170/5170	6288/6288	1119/1119	−26/−26	ATA/ATA	TAG/TAG	N/N
*trnS2*	6291/6291	6350/6351	60/61	2/2			N/N
*CR*	6351/6352	7518/7505	1168/1154				/
*trnH*	7519/7506	7581/7568	63/63				N/N
*rrnS*	7703/7690	8439/8426	737/737	121/121			N/N
*rrnL*	8450/8437	9809/9795	1360/1359	10/10			N/N
*trnL1*	9799/9786	9858/9844	60/59	−11/−11			N/N
*ND1*	9859/9845	10,786/10,772	928/928		ATA/GTA	T/T	N/N
*trnP*	10,787/10,773	10,849/10,835	63/63				N/N
*ND4L*	10,851/10,837	11,132/11,118	282/282	1/1	ATG/ATG	TAG/TAG	N/N
*ND4*	11,222/11,208	12,457/12,443	1236/1236	89/89	ATG/ATG	TAG/TAG	N/N
*trnT*	12,458/12,444	12,523/12,509	66/66				N/N
*ND5*	12,665/12,650	14,354/14,339	1690/1690	141/141	ATG/ATA	T/T	N/N
*trnF*	14,364/14,349	14,425/14,410	62/62	9/9			N/N
*trnY*	14,426/14,411	14,488/14,473	63/63				N/N
*trnQ*	14,500/14,485	14,562/14,547	63/63	11/11			N/N
*trnC*	14,563/14,548	14,624/14,609	62/62				N/N
*trnM*	14,679/14,664	14,742/14,727	64/64	54/54			N/N
*ND2*	14,752/14,737	15,747/15,732	996/996	9/9	ATT/ATA	TAG/TAG	N/N
*trnW*	15,748/15,733	15,813/15,798	66/66				N/N

**Table 3:** Base compositions of the whole genomes, PCGs, rRNAs, and tRNAs of the six Polydesmida mitogenomes.

Region	Species	Length (bp)	A + T%	AT-Skew	GC-Skew
Whole mitogenome	red *Nedyopus patrioticus*	15,834	68.23	−0.264	0.462
	white *Nedyopus patrioticus*	15,798	68.28	−0.262	0.463
	*Xystodesmus* sp. YD-2016	15,791	67.01	−0.217	0.471
	*Asiomorpha coarctata*	15,644	67.45	−0.235	0.429
	*Epanerchodus koreanus*	15,581	75.11	−0.260	0.446
	*Appalachioria falcifera*	15,282	64.04	−0.368	0.441
PCGs	red *Nedyopus patrioticus*	10,767	67.93	−0.346	0.464
	white *Nedyopus patrioticus*	10,767	67.00	−0.344	0.464
	*Xystodesmus* sp. YD-2016	10,995	65.57	−0.301	0.460
	*Asiomorpha coarctata*	11,019	66.11	−0.331	0.428
	*Epanerchodus koreanus*	10,959	73.87	−0.357	0.435
	*Appalachioria falcifera*	10,998	63.08	−0.447	0.451
rRNAs	red *Nedyopus patrioticus*	2097	72.53	−0.073	0.434
	white *Nedyopus patrioticus*	2096	72.62	−0.074	0.446
	*Xystodesmus* sp. YD-2016	2007	69.20	−0.042	0.511
	*Asiomorpha coarctata*	2016	69.34	−0.044	0.421
	*Epanerchodus koreanus*	2082	79.06	−0.066	0.450
	*Appalachioria falcifera*	2025	68.75	−0.221	0.472
tRNAs	red *Nedyopus patrioticus*	1203	67.67	−0.091	0.414
	white *Nedyopus patrioticus*	1208	67.31	−0.082	0.428
	*Xystodesmus* sp. YD-2016	1430	71.05	−0.069	0.338
	*Asiomorpha coarctata*	1437	69.52	−0.083	0.361
	*Epanerchodus koreanus*	1378	77.14	−0.091	0.435
	*Appalachioria falcifera*	1363	66.47	−0.157	0.343

## Data Availability

DNA sequences: GenBank accession number OR755973 for red *Nedyopus patrioticus* and OR777861 for white *Nedyopus patrioticus*.
